# Proteome analysis guided genetic engineering of *Corynebacterium glutamicum* S9114 for tween 40-triggered improvement in l-ornithine production

**DOI:** 10.1186/s12934-019-1272-0

**Published:** 2020-01-06

**Authors:** Yan Jiang, Ming-Zhu Huang, Xue-Lan Chen, Bin Zhang

**Affiliations:** 1 College of Bioscience and Engineering, Jiangxi Engineering Laboratory for the Development and Utilization of Agricultural Microbial Resources, Jiangxi Agricultural University, Nanchang, 330045 China; 2 College of Life Science, Jiangxi Normal University, Nanchang, 330022 China

**Keywords:** *Corynebacterium glutamicum*, Tween 40, l-ornithine, Metabolic engineering

## Abstract

**Background:**

l-ornithine is a valuable amino acid with a wide range of applications in the pharmaceutical and food industries. However, the production of l-ornithine by fermentation cannot compete with other methods, because of the low titers produced with this technique. Development of fermentation techniques that result in a high yield of l-ornithine and efficient strategies for improving l-ornithine production are essential.

**Results:**

This study demonstrates that tween 40, a surfactant promoter of the production of glutamate and arginine, improves l-ornithine production titers in engineered *C. glutamicum* S9114. The intracellular metabolism under tween 40 triggered fermentation conditions was explored using a quantitative proteomic approach, identifying 48 up-regulated and 132 down-regulated proteins when compared with the control. Numerous proteins were identified as membrane proteins or functional proteins involved in the biosynthesis of the cell wall. Modulation of those genes revealed that the overexpression of *CgS9114_09558* and the deletion of *CgS9114_13845*, *CgS9114_02593,* and *CgS9114_02058* improved the production of l-ornithine in the engineered strain of *C. glutamicum* Orn8. The final strain with all the exploratory metabolic engineering manipulations produced 25.46 g/L of l-ornithine, and a yield of 0.303 g l-ornithine per g glucose, which was 30.6% higher than that produced by the original strain (19.5 g/L).

**Conclusion:**

These results clearly demonstrate the positive effect of tween 40 addition on l-ornithine accumulation. Proteome analysis was performed to examine the impact of tween 40 addition on the physiological changes in *C. glutamicum* Orn8 and the results showed several promising modulation targets for developing l-ornithine-producing strains.

## Background

l-ornithine is a valuable non-protein amino acid that is an intermediate metabolite involved in the urea cycle. It has numerous biological functions such as the treatment of liver disease, the promotion of wound healing, and it also plays a role in the anti-aging process [1]. Due to its various advantages in human physiological functions and widespread application in the pharmaceutical and food industries, the global l-ornithine market has seen rapid development in recent years [2]. Currently, the industrial production of l-ornithine is primarily depending on enzyme-catalyzed arginine hydrolysis and microbial fermentation [3]. Developing an efficient microbial cell factory to produce l-ornithine is a promising approach owing to its advantages in low-cost, sustainability, and environment friendly. Regarding current work in this field, it is important to point out that there are increasing in number of engineered strains, such as *Escherichia coli* [4], *Saccharomyces cerevisiae* [5], and *Corynebacterium glutamicum* [6], that have been modulated for l-ornithine production. *E. coli* is a model microbe with the advantages of having an explicit genetic background, a superior propagation speed, and convenient gene manipulation tools that have been employed for producing l-ornithine by rational modulation of the urea cycle and optimizing the fermentation process [4]. However, *E. coli* fermentation inevitably results in the synthesis of endotoxin [7], a toxic substance that limits its application in the fermentation production of l-ornithine. *S. cerevisiae* is a superior eukaryotic microbial cell factory that has been defined as a ‘Generally Recognized As Safe’ (GRAS) strain which exhibits a high tolerance to harsh growing conditions and can be used to produce numerous valuable chemicals [8–10]. Modularized metabolic engineering strategies including the transformation of the carbon source transport system, the transformation of the central metabolic pathway, the assimilation and dissimilation of ammonia, and the energy supply and transfer of small molecular compounds were all processed in *S. cerevisiae* to produce l-ornithine [5]. However, the obtained production titer of l-ornithine by using *S. cerevisiae* as chassis microorganism is still in the preliminary stage and far away from the requirements of industrialization. For producing l-ornithine, *C. glutamicum* occupies the dominant position [11].

*Corynebacterium glutamicum* is a promising GRAS gram-positive bacterium that was intensively engineered to utilize a broad spectrum of carbon sources for the overproduction of numerous chemicals [12–14]. Currently, several studies have attempted to develop metabolically engineered strains that are able to rapidly convert a certain amount of glucose, or other alternative carbon resource, into l-ornithine. The biosynthesis pathway of l-ornithine in *C. glutamicum* is described in previous studies [15–18]. Jensen et al. [19] developed an l-ornithine producing *C. glutamicum* strain ORN6 through rational engineering, including the deletion of *argF*, *argR*, and *argG*, enhancing the supplement of glutamate via the optimized expression of *gdh*, redirection of the metabolic flux to the pentose phosphate pathway to provide adequate cofactor NADPH via a change of the start codon of *pgi*, overexpression of a feedback insensitive N-acetylglutamate kinase, and the insertion of a second copy of the *argCJB*^*M*^*D* operon into the chromosome, resulting in the production of l-ornithine with a yield of 0.52 g of l-ornithine per g glucose. Kim et al. [6] constructed an engineered *C. glutamicum* strain through the deletion of *argF*, *proB*, and *argR*, plasmid-based overexpression of the *argCJBD* genes from *C. glutamicum* ATCC 21831, as well as the redirection of the carbon flux towards the pentose phosphate pathway via the modulated expression of *pgi*, *zwf*, and *tkt* operons that produced 51.5 g/L of l-ornithine with a yield of 0.256 g/g glucose in a 6.6-L fermenter. Jiang et al. [20] created an engineered strain by systematically manipulating l-ornithine metabolism and adaptive evolution that produced 24.1 g/L of l-ornithine in a 5-L bioreactor. Shu et al. [21] performed an experiment that involved blocking the competing branch of the l-ornithine synthesis pathway, the site-directed mutagenesis of ornithine acetyltransferase, and the heterologous overexpression of the genes *argA* from *E. coli* and *argE* from *Serratia marcescens* in an l-arginine hyper-producing strain of *Corynebacterium crenatum.* This resulted in an engineered strain of Cc-QF-4, which produced 40.4 g/L of l-ornithine with a yield of 0.269 g/g glucose in a 5-L bioreactor.

Polyoxyethylene sorbitan monopalmitate (Tween 40), a fatty acid ester surfactant, is widely employed as a fermentation trigger for industrial fermentation [22]. In *C. glutamicum*, the addition of tween 40 cannot only redirect the carbon flux towards the biosynthesis of glutamate by reducing the enzyme activity of α-ketoglutarate dehydrogenase, but can also promote the secretion of glutamate by enhancing membrane permeability [23]. Addition of tween 40 is able to change the rigid membrane caused by inducement of excess biotin, a critical factor affecting the performance of glutamate fermentation in biotin auxotrophic *C. glutamicum*, which stimulate glutamate overproduction. In addition, the effect of fermentation promotion by tween 40 has been assessed in biotin auxotrophic *C. glutamicum* for the production of several products including L-glutamate, l-arginine [24], gamma-amino butyric acid [25], oleic acid [26], and 1,5-diaminopentane [27], whereas no studies have reported the effect of tween 40 on l-ornithine fermentation.

In a previous study, we systematically developed an engineered strain of *C. glutamicum* S9114 through the deletion of *argF, ncgl1221, argR,* and *putP,* the attenuation of *odhA*, and the overexpression of *argCJBD*, which produced up to 19 g/L of l-ornithine in a shaker flask culture [15, 16]. In the present study, the effects of tween 40 on l-ornithine production were investigated. In addition, differentially expressed proteins, triggered by adding tween 40, were identified using a total proteomics analysis. Furthermore, the effects of overexpressing some of the upregulated proteins and deleting a few of the downregulated proteins on l-ornithine accumulation were evaluated.

## Results

### Effect of tween 40 on l-ornithine production in *C. glutamicum*

**Fig. 1:**
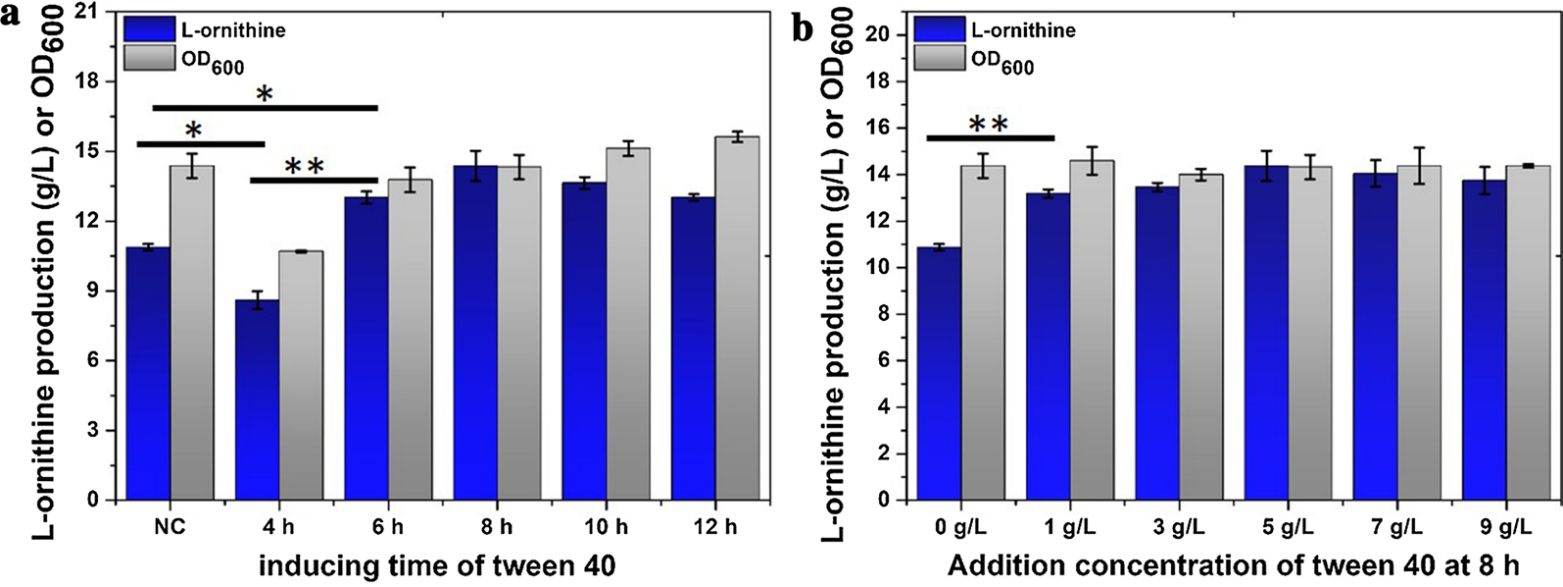
Effect of tween 40 addition on cell growth and l-ornithine production. **a** Optimizing the temporal points of tween 40 addition. **b** Optimizing tween 40 addition concentration. Gray bar represents OD_600_, blue bar represents l-ornithine concentration. Results of standard deviations present in three individual experiments. ^*^P < 0.05, ^**^P < 0.01

Tween 40 is a fermentation accelerator that is commonly used to produce l-glutamate in *C. glutamicum* when excessive biotin leads to an impermeable cell membrane. As previously reported, addition of tween 40 during the logarithmic period cannot only induce glutamate overproduction, but can also improve l-arginine production in *C. crenatum* by reforming cell surface structures and reducing the enzyme activity of α-oxoglutarate dehydrogenase. Theoretically, addition of tween 40 can promote the biosynthesis of l-ornithine by redirecting the carbon flux from the tricarboxylic acid cycle to glutamate accumulation or improving membrane permeability in *C. glutamicum*. To further characterize the effect of tween 40 on l-ornithine production, we optimized the addition concentration and addition phase during fermentation of the strain *C. glutamicum* Orn8. Results after 40 h of *C. glutamicum* Orn8 fermentation using a shake flask suggests that the production titer was significantly affected by the temporal points of filtration degermed tween 40 addition, and cell growth was also reduced when the temporal points of filtration degermed tween 40 addition was less than 6 h (Fig. 1a). The yield of l-ornithine was not further improved by optimizing the addition concentration of tween 40 from 1 g/L to 9 g/L (Fig. 1b). Consequently, the highest l-ornithine production titer (14.4 ± 0.64) was obtained by using an 8 h temporal points of tween 40 addition and an addition concentration of 5 g/L, which is 33.3% higher than that of the control without the addition of tween 40 (10.8 g/L). The significance of this finding rests on the fact that the addition of Tween 40 significantly improved l-ornithine production in the engineered strain of *C. glutamicum* Orn8.

### MS identification of differentially expressed proteins induced by tween 40

**Fig. 2:**
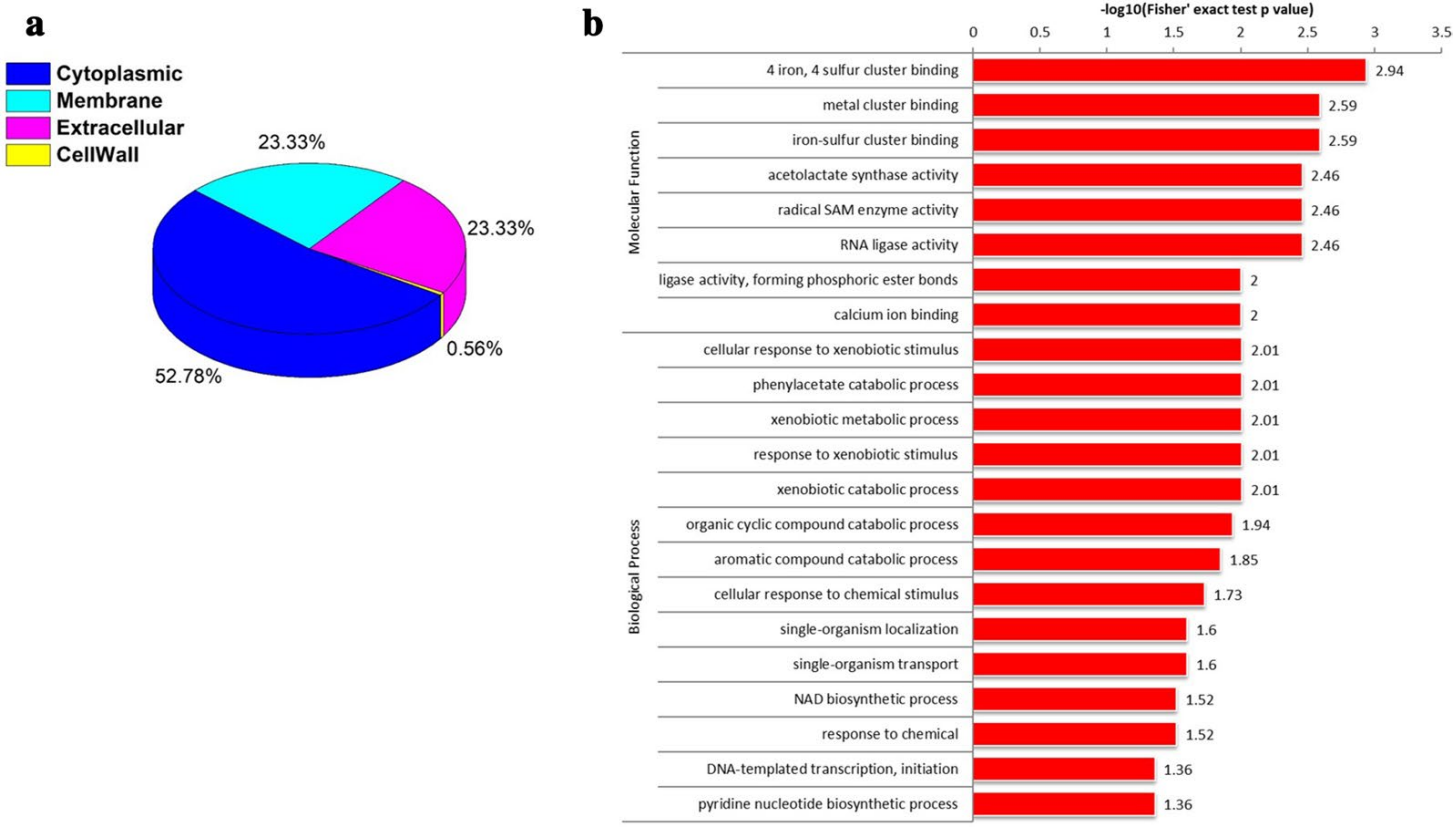
Subcellular localization and gene ontology enrichment analysis of different expression protein identified by proteomics approach. **a** Subcellular localization pie chart. **b** Two-tailed Fisher’s exact test enrichment analysis of the differentially expressed protein. The gene ontology with a corrected P-value < 0.05 was considered significant. This filtered P value matrix was transformed by the function x = − log10 (P value). Samples were collected at 12 h for total proteomics analysis after treated by tween 40

**Fig. 3:**
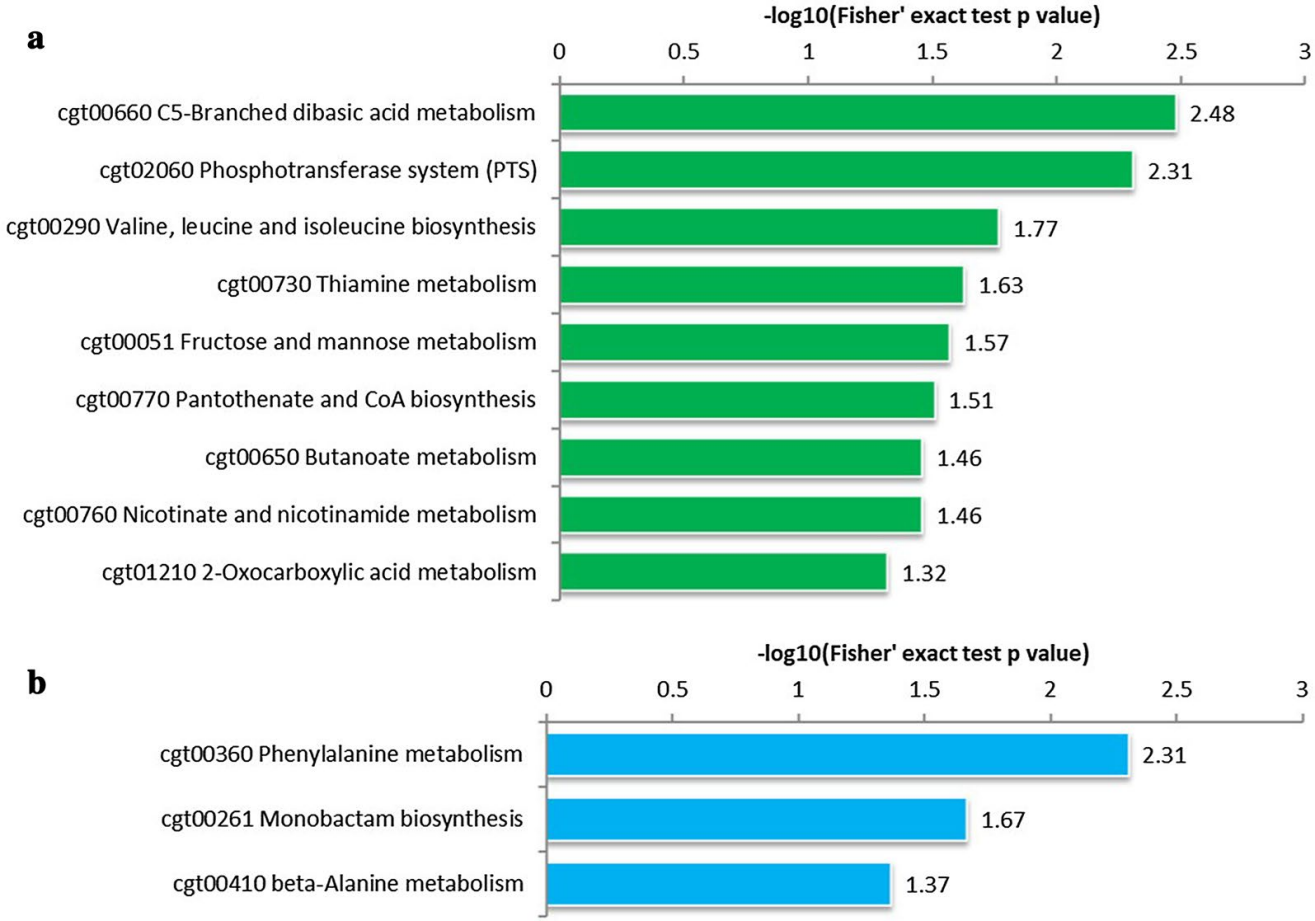
Pathway enrichment analysis of the differentially expressed protein. **a** Pathway enrichment analysis of upregulated proteins. **b** Pathway enrichment analysis of downregulated proteins. The pathway with a corrected P-value < 0.05 was considered significant. This filtered P value matrix was transformed by the function x = − log10 (P value)

Although the addition of tween 40 can increase the production titer of l-ornithine, the specific mechanisms of action in strain *C. glutamicum* Orn8 have not been fully elucidated. To gain further insight into the involvement of adding tween 40 during the logarithmic phase on the physiological mechanisms of *C. glutamicum* Orn8, a quantitative proteomics assay using stable isotope dimethyl labeling was processed to identify the up and down regulated proteins. Three biological repetitions were employed to ensure the accuracy of the experimental data in differentially expressed proteins. A total of 2237 proteins were identified in this study, of which 2138 contained quantitative information (Additional file 1). If 1.2-fold was taken as the minimum change threshold and a *t* test p-value < 0.05 as the standard, 180 proteins were recognized with expression alternation from mass spectrometry identification results (Additional file 2). Among them, 48 proteins were overexpressed, whereas 132 proteins were downregulated after the cell was exposed to tween 40 in the engineered strain of *C. glutamicum* Orn8. The subcellular location analysis of these differentially expressed proteins suggests that 95 proteins (52.78%) were functioning in the cytoplasm, 42 proteins (23.33%) were functioning on the cell membrane, 42 proteins (23.33%) were functioning in the extracellular matrix, and only one protein functioned in the cell wall (Fig. 2a). Subsequently, the molecular functions of these differentially expressed proteins were classified by GO annotations, and are mainly involved in 4 iron, 4 sulfur cluster binding, metal cluster binding, acetolactate synthase activity, radical SAM enzyme activity, ligase activity, forming phosphoric ester bonds, and calcium ion binding (Fig. 2b). Particularly, the expression of *ncgl1273* (*dtsR2*), encoding an acetyl-CoA carboxylase biotin carboxyl carrier protein subunit, was downregulated by 1.22-fold, and the expression of *CgS9114_13431* (*dtsR1*), encoding an acetyl-CoA carboxyltransferase, was downregulated by 1.66-fold, suggesting that the fatty acid synthesis pathway was weakened by the addition of tween 40 in the strain of *C. glutamicum* Orn8. In addition, the biological processes of these differentially expressed proteins were mainly involved in the cellular response to xenobiotic stimulus, the phenylacetate catabolic process, the organic cyclic compound catabolic process, the aromatic compound catabolic process, the cellular response to chemical stimulus, the NAD biosynthetic process, and the pyridine nucleotide biosynthetic process. Furthermore, results of KEGG enriched analysis indicated that the cytoplasmic upregulated proteins were mainly listed on the metabolic pathway of C5-branched dibasic acid metabolism, the phosphotransferase system, branched amino acid biosynthesis, thiamine metabolism, fructose and mannose metabolism, pantothenate and CoA biosynthesis, butanoate metabolism, nicotinate and nicotinamide metabolism, and 2-oxocarboxylic acid metabolism (Fig. 3a). The downregulated proteins were mainly involved in phenylalanine metabolism, monobactam biosynthesis, and β-alanine metabolism (Fig. 3b). Interestingly, *bioB* encoding a biotin synthase, *bioN* encoding a biotin transport system permease protein, and *bioM* encoding a biotin transport system ATP-binding protein were attenuated by 1.22, 1.30, and 1.33-fold, respectively, which indicated that the biosynthesis pathway of biotin was affected by the presence of tween 40. Detailed protein information is presented in the supporting materials (Additional files 1, 2) Taken together, these differentially expressed proteins triggered by the addition of tween 40 cover a wide range of metabolic and physiological processes.

### Effect of overexpressing upregulated membrane proteins induced by tween 40 addition on l-ornithine production

**Fig. 4:**
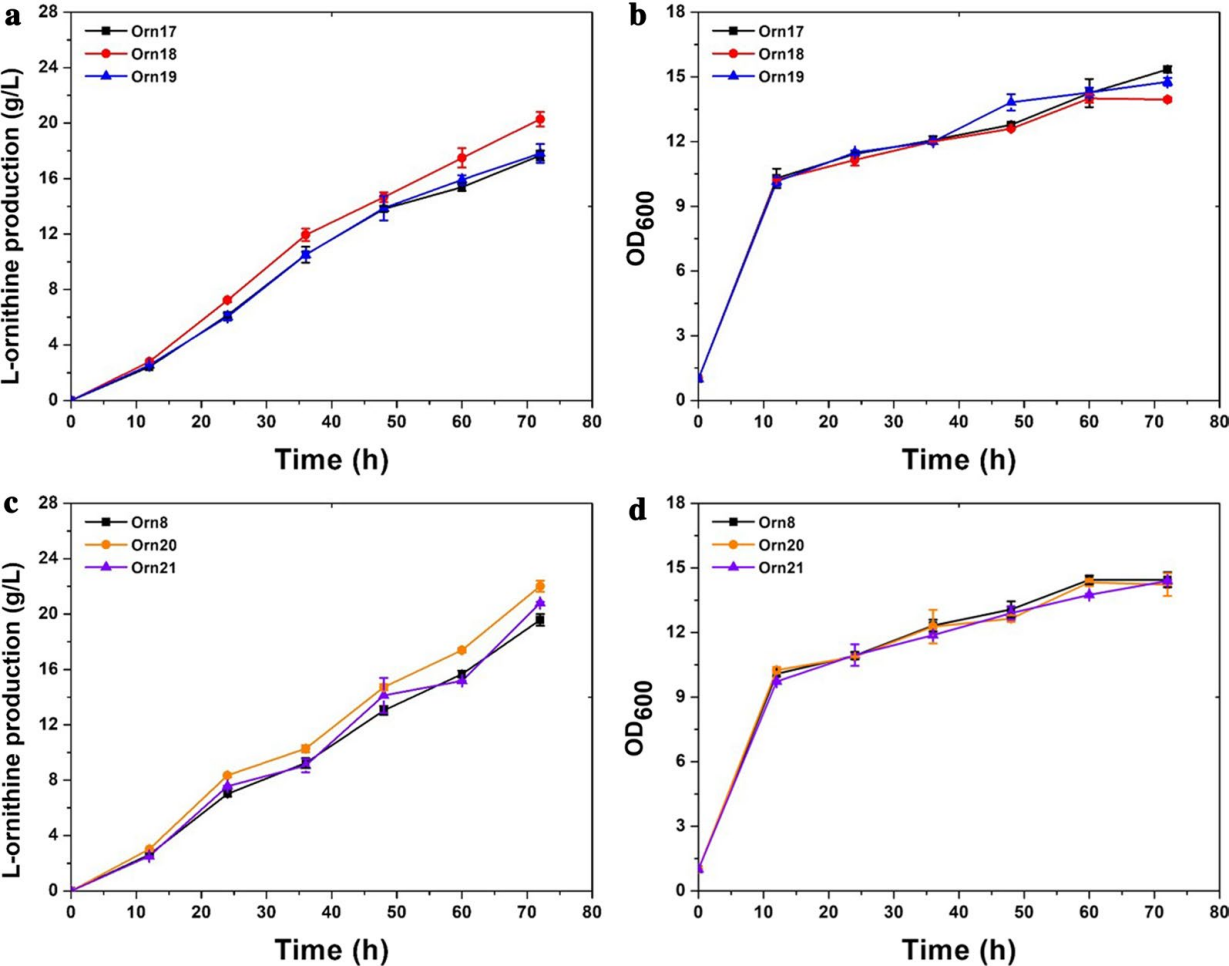
Assessment of cell growth and ʟ-ornithine productivity of strains Orn8, Orn17(Orn8 carrying empty plasmid pEC-XK99E), Orn18(Orn8 carrying expression plasmid pEC-*CgS09558*), Orn19 (Orn8 carrying expression plasmid pEC-*CgS14252*), Orn20 (Orn8 carrying P_*tac*_ promoter inserted in the upstream region of *CgS9114_09558*), Orn21(Orn8 carrying P_*tac*_ promoter inserted in the upstream region of *CgS9114_14252*). **a** ʟ-ornithine production curves for strains Orn17, Orn18, and Orn19. **b** Cell growth curves for strains Orn17, Orn18, and Orn19. **c** ʟ-ornithine production curves for strains Orn8, Orn20, and Orn21. **b** Cell growth curves for strains Orn8, Orn20, and Orn21. Samples were collected per 12 h for fermentation parameter determination. Data represent average values and standard deviations from three individual experiments

As previously demonstrated, tween 40 addition reduced α-oxoglutarate dehydrogenase by downregulating the expression of *dtsR1*, a subunit of acetyl-CoA carboxylase, which improved the production titer of l-arginine by 16.5% in *C. crenatum* [24]. Theoretically, attenuation of acetyl-CoA carboxylase exhibits three functional mechanisms for glutamate-derived compounds overproduction, such as reducing the α-oxoglutarate dehydrogenase activity that converts the metabolic fluxes into the l-glutamate biosynthetic pathway, providing more cofactor acetyl-CoA for the biosynthesis of N-acetylglutamate by attenuating the conversion from acetyl-CoA to malonyl-CoA, and improving membrane permeability by reducing the biosynthesis of phospholipids. From the results of a quantitative proteomics assay, *dtsR1* was identified and downregulated by tween 40 addition. To further substantiate the role of *dtsR1* on l-ornithine accumulation, we attempted to attenuate its expression using gene deletion, an RBS change, the insertion of a terminator, and by adding a C-terminal degradation label of ASV or AAV in the strain of *C. glutamicum* Orn8 by using a homologous recombination technique. Unfortunately, no positive recombinant strains were found on the plate, suggesting that *dtsR1* is necessary for cell growth and cannot be weakened in *C. glutamicum* Orn8 (data not shown). The original aim of this study was to find efficient targets for improving l-ornithine production. Thus, we attempted to overexpress proteins that were upregulated by more than two-fold compared to the control (shown in Table 1). An *E. coli*-*C. glutamicum* shuttle expression plasmid pEC-XK99E was used for the expression of *CgS9114_09563*, *CgS9114_14252*, *CgS9114_09558*, *CgS9114_04075*, and *CgS9114_11142*. However, only pEC-*CgS14252* and pEC-*CgS09558* were successfully overexpressed in the strain of *C. glutamicum* Orn8, and thus generated the strains Orn18 and Orn19. Overexpression of the other three genes was probably toxic to the cell and led to mutant clones that were not viable. The empty plasmid pEC-XK99E was also introduced to *C. glutamicum* Orn8 and generated strain Orn17 as control. Result of 72 h shake flask cultivation revealed that strain Orn19 produced 20.28 ± 0.5275 g/L of l-ornithine which is 15% higher than that of the control strain Orn17(17.66 ± 0.3587), and strain Orn18 produced an approximately equal amount of l-ornithine (17.81 ± 0.6752) as compared with the control strain Orn17 (Fig. 4a; Table 2). In addition, the cell growth was not affected by the plasmid-based overexpression of *CgS9114_14252* and *CgS9114_09558* (Fig. 4b). To further elucidate the role of *CgS9114_14252* and *CgS9114_09558* on l-ornithine accumulation, we inserted a strong P_*tac*_ promoter in the upstream region of each gene, which resulted in strains Orn20 and Orn21. As expected, strain Orn20 harboring *CgS9114_09558* overexpression produced 12.8% more l-ornithine than that of the control strain Orn8, while strain Orn21 produced an equal concentration of l-ornithine as the control strain Orn8 (Fig. 4c, d). Taken together, these results illustrate that overexpression of *CgS9114_09558* promotes l-ornithine production in mutant *C. glutamicum* Orn8, although the biological function has not been fully elucidated.

**Table 1:** Two-fold above differentially expressed proteins identified by bioinformatic analysis

Uniprot no.	Name	Description	Fold change	P-value	Regulated type	Protein MW [kDa]
UPI0001335099	*CgS9114_09563*	Membrane protein	7.256	1.4154E-06	Up	30.261
UPI0000165D0A	*CgS9114_14252*	Thiamin-regulated hydroxymethylpyrimidine ECF transporter	4.397	0.00076343	Up	21.047
UPI0001335098	*CgS9114_09558*	ABC transporter	3.364	0.00023894	Up	33.102
UPI0002230CC7	*CgS9114_04075*	Sugar/inositol transporter	2.42	0.0037017	Up	53.663
UPI00000B98E2	*CgS9114_11142*	RNA polymerase sigma-70	2.285	0.00149636	Up	37.573
UPI00013356FF	*CgS9114_11567*	Hypothetical protein	− 2	0.00016463	Down	12.641
UPI0002233099	*CgS9114_12732*	Hypothetical protein	− 2.079	0.035118	Down	19.917
UPI000003A2AE	*CgS9114_13171*	Transcription factor WhiB	− 2.141	1.96915E−05	Down	9.5859
UPI0002233056	*CgS9114_12442*	Peptidoglycan recognition protein	− 2.188	2.4755E−05	Down	71.159
UPI0002232B52	*CgS9114_00255*	Calcium ion binding	− 2.32	2.6369E−06	Down	90.876
UPI000223314F	*CgS9114_13516*	Hypothetical protein	− 2.32	0.00036372	Down	17.644
UPI0002230CB5	*CgS9114_01180*	Hypothetical protein	− 2.387	2.155E−07	Down	79.258
UPI000223195D	*CgS9114_04917*	Hypothetical protein	− 2.5	8.4307E−05	Down	18.129
UPI000223312F	*CgS9114_13366*	Hypothetical protein	− 2.584	0.0026047	Down	18.208
UPI00013350CF	*CgS9114_09908*	Putative esterase	− 2.611	3.4887E−06	Down	43.563
UPI0002230695	*CgS9114_09663*	Hypothetical protein	− 2.653	0.00010412	Down	13.101
UPI00022330EE	*CgS9114_13056*	Peptidase S1	− 2.924	1.99298E−05	Down	36.882
UPI0002230696	*CgS9114_09668*	Hypothetical protein	− 2.967	0.00058298	Down	13.194
UPI000223195B	*CgS9114_04907*	Hypothetical protein	− 3.058	0.00129914	Down	18.18
UPI000003A50D	*CgS9114_14497*	Putative esterase	− 3.378	3.5667E−05	Down	33.531
UPI0002231962	*CgS9114_04952*	Putative esterase	− 3.401	2.0229E−08	Down	39.62
UPI000133536B	*CgS9114_13715*	Putative secreted protein	− 3.413	2.1044E−08	Down	29.955
UPI000133510C	*CgS9114_14352*	Hypothetical protein	− 3.521	0.00018473	Down	24.492
UPI0001335576	*CgS9114_00220*	Ferredoxin-NADP+ reductase	− 3.597	5.1049E−07	Down	50.056
UPI000223193A	*CgS9114_01878*	protein prenyltransferase	− 3.774	6.0916E−07	Down	40.614
UPI0002231C7B	*CgS9114_02058*	l, d-transpeptidase	− 3.817	3.7351E−07	Down	26.282
UPI0002232E3D	*CgS9114_06260*	Hypothetical protein	− 3.953	3.3347E−09	Down	18.827
UPI0002231960	*CgS9114_04942*	Calcium-binding	− 4.149	0.00013962	Down	18.34
UPI00022322E5	*CgS9114_02593*	Lipocalin	− 4.762	3.0099E−06	Down	23.181
UPI00013353A8	*CgS9114_13845*	Peptidase	− 6.494	5.3207E−07	Down	24.359
UPI0002231622	*CgS9114_01853*	Hypothetical protein	− 8.065	1.6529E−07	Down	21.665

**Table 2:** Engineering the l-ornithine production in recombinant *C. glutamicum*

Strains	Cell biomass (OD_600_)	l-ornithine accumulation (g/L)	l-ornithine/cell biomass (OD_600_)
orn8	14.45 ± 0.35	19.58 ± 0.42	1.36
orn17	15.35 ± 0.15	17.66 ± 0.36	1.15
orn18	13.95 ± 0.10	20.28 ± 0.53	1.45
orn19	14.78 ± 0.18	17.81 ± 0.68	1.21
orn20	14.23 ± 0.53	22.01 ± 0.40	1.55
orn21	14.40 ± 0.05	20.79 ± 0.11	1.44
orn22	15.85 ± 0.75	22.52 ± 0.49	1.42
orn23	15.08 ± 0.73	21.86 ± 0.08	1.45
orn24	15.45 ± 0.05	21.84 ± 0.91	1.41
orn25	14.20 ± 0.35	18.87 ± 0.55	1.33
orn26	14.33 ± 0.28	17.18 ± 0.17	1.20
orn27	14.35 ± 0.60	16.93 ± 0.42	1.18
orn28	14.88 ± 0.03	23.83 ± 0.19	1.60
orn29	14.58 ± 0.13	25.46 ± 0.23	1.75
Orn30	15.43 ± 0.28	25.88 ± 0.79	1.68

Fermentations were performed at 250 rpm for 72 h, and the initial glucose concentration was 100 g/L

Results are the means ± standard deviations in three individual experiments

### Improvement of l-ornithine production by the deletion of the downregulated proteins

**Fig. 5:**
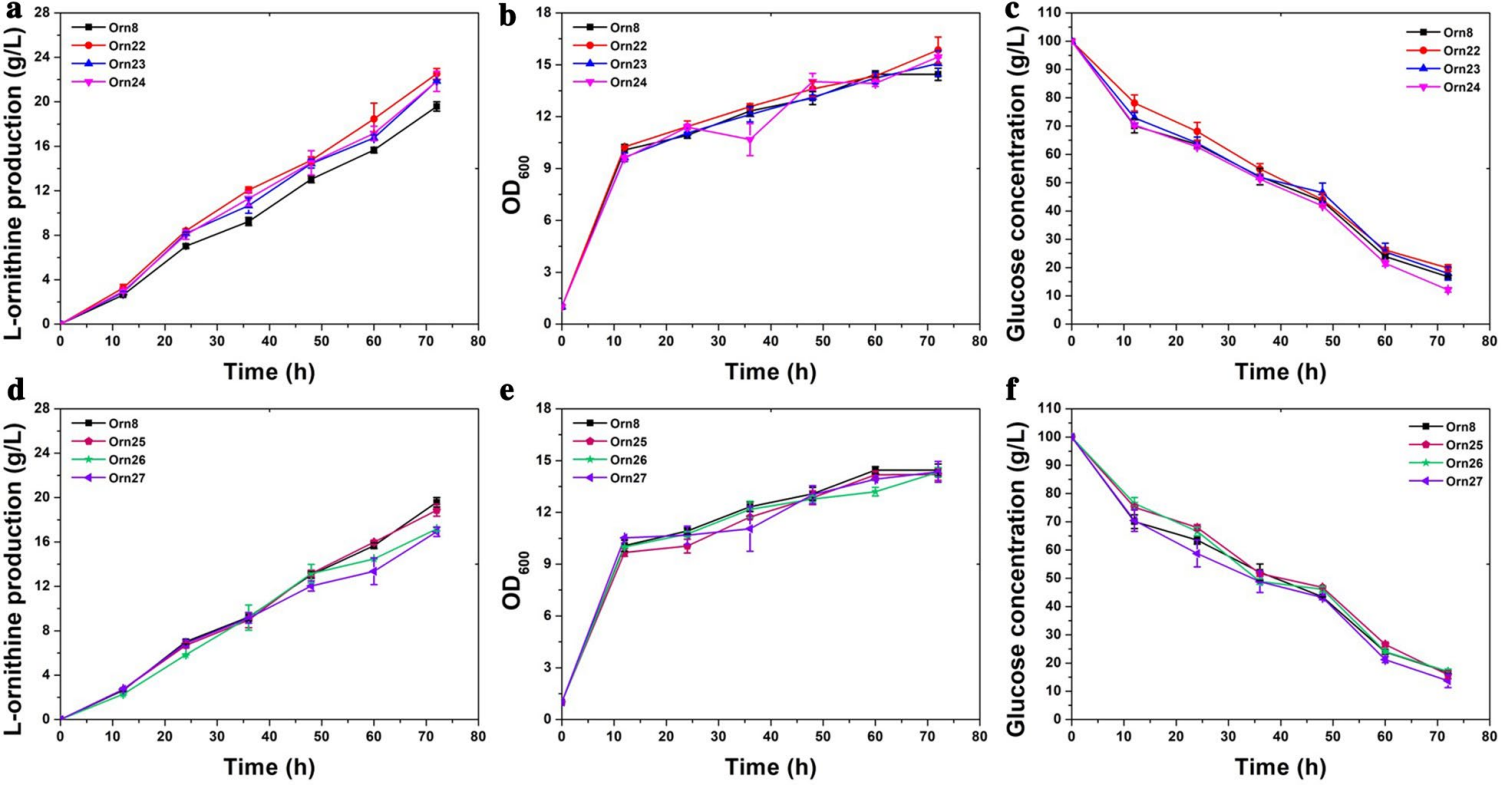
Measurement of cell growth, ʟ-ornithine productivity, and glucose consumption performed by strain Orn8, Orn22 (Orn8 with deletion of *CgS9114_13845*), Orn23 (Orn8 with deletion of *CgS9114_02593*), Orn24 (Orn8 with deletion of *CgS9114_02058*), Orn25 (Orn8 with deletion of *CgS9114_14352*), Orn26 (Orn8 with deletion of *CgS9114_04952*), Orn27 (Orn8 with deletion of *CgS9114_14497*). **a** ʟ-ornithine production curves for strains Orn8 (black square), Orn22 (red circle), Orn23 (blue upper triangle), Orn24(pink lower triangle). **b** Cell growth of strain Orn8, Orn22, Orn23, and Orn24. **c** Residual glucose concentration curves of strain Orn8, Orn22, Orn23, and Orn24. **d** ʟ-ornithine production curves for strains Orn8 (black square), Orn25 (dull-red pentagon), Orn26 (green pentacle), Orn27(purple left triangle). Samples were collected per 12 h for fermentation parameter determination. Data represent the average values and standard deviations from three individual experiments

**Fig. 6:**
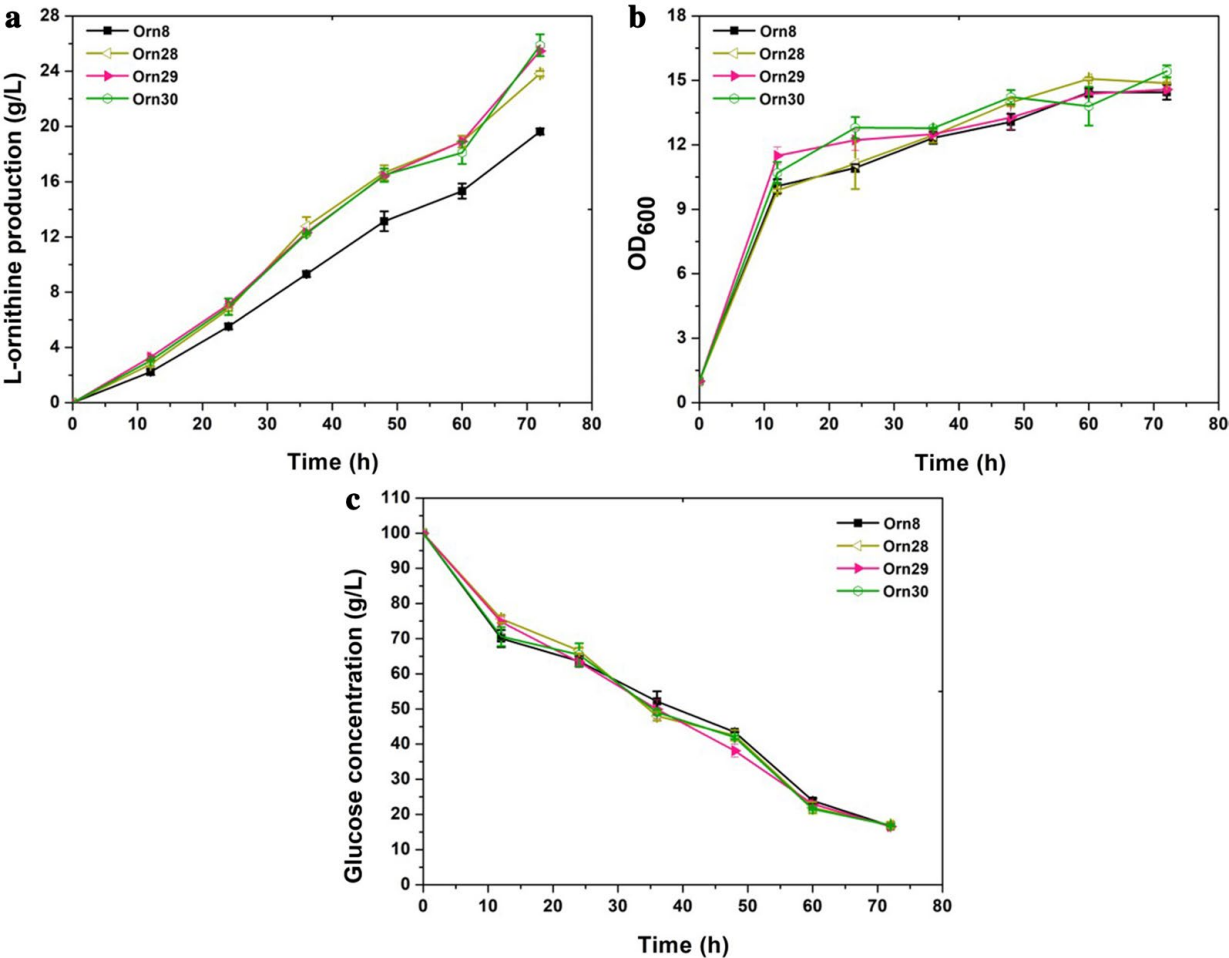
Effect of combined modulating *CgS9114_13845*, *CgS9114_02593*, *CgS9114_02058*, and *CgS9114_09558* on l-ornithine production. **a** ʟ-ornithine production curves for strains Orn8 (black square), Orn28 (yellow left triangle) (Orn8 with deletion of *CgS9114_13845* and *CgS9114_02593*), Orn29 (red right triangle) (Orn8 with deletion of *CgS9114_13845*, *CgS9114_02593* and *CgS9114_02058*), Orn30 (green cycle) (Orn8 with deletion of *CgS9114_13845*, *CgS9114_02593*, and *CgS9114_02058*; overexpression of *CgS9114_09558*).**b** cell growth of those strains. **c** Residual glucose concentration curves of those strain. Samples were collected per 12 h for measuring fermentation parameter. Data represent the average values and standard deviations from three individual experiments

As can be seen in Table 1, the addition of tween 40 not only stimulated the expression of *CgS9114_09563*, *CgS9114_14252*, *CgS9114_09558*, *CgS9114_04075*, and *CgS9114_11142*, but also inhibited the expression of numerous other genes. To further characterize the effect of these downregulated genes on l-ornithine production, we examined eight genes, including *CgS9114_13845*, *CgS9114_02593*, *CgS9114_02058*, *CgS9114*_*14352*, *CgS9114_04952*, *CgS9114_14497*, *CgS9114_01853*, and *CgS9114_04942*, which had the highest expression fold changes, using gene deletion assays. Among them, only six genes were successfully deleted, which generated the recombinant strains Orn22, Orn23, Orn24, Orn25, Orn26, and Orn27. As shown in Fig. 5a, d, strain Orn22 with the deletion of *CgS9114_13845*, Orn23 with the deletion of *CgS9114_02593*, and Orn24 with the deletion of *CgS9114_02058* produced 22.51 ± 0.4853, 21.86 ± 0.0844, and 21.8421 ± 0.9073 g/L of l-ornithine after 72 h of shake flask cultivation respectively, which is 15%, 11.6%, and 11.5% higher than that (19.5 g/L) of the original strain, Orn8 (Table 2). Nevertheless, strain Orn25 with the deletion of *CgS9114_14352*, Orn26 with the deletion of *CgS9114_04952*, and Orn27 with the deletion of *CgS9114_14497* produced 18.87 ± 0.5486, 17.18 ± 0.1688, 16.93 ± 0.422 g/L of l-ornithine respectively, which exhibits disparate degrees of decline in l-ornithine production titer when compared with the control strain, Orn8 (Table 2). Fortunately, the deletion of those genes exerts no obvious disturbance on cell growth or glucose consumption (Fig. 5b, c, e, f). In addition, the synergistic effect of three positive targets was also investigated using combinational modulation, which resulted in strain Orn28, Orn29, and Orn30. As illustrated in Fig. 6a, an engineered strain of Orn28 harboring a double deletion of *CgS9114_13845* and *CgS9114_02593* produced 23.83 ± 0.1892 g/L of l-ornithine with a yield of 0.287 g of ornithine per g of glucose (Table 2). Furthermore, Orn29 with a triple deletion of *CgS9114_13845*, *CgS9114_02593,* and *CgS9114_02058* produced 25.46 ± 0.2270 g/L of l-ornithine with a yield of 0.303 g of ornithine per g of glucose, which is 23% and 31% higher than that of the control strain of Orn8, respectively (Table 2). Combination modulation of these deletion targets exhibited an efficient synergistic effect on improving the yield of l-ornithine in strain Orn8 and exerts no negative effect on cell growth or glucose consumption (Fig. 6b, c). However, overexpression of *CgS9114_09558* in strain Orn29 generated strain Orn30, and was not able to further promote l-ornithine accumulation. In conclusion, these results suggest that modulation of these targets derived from proteomics exhibit a positive synergistic effect on the biosynthesis of l-ornithine in *C. glutamicum*.

## Discussion

In this study, we attempted a system-wide demonstration of the effect of adding tween 40 on l-ornithine production and examined its physiological mechanism in *C. glutamicum* Orn8. As anticipated, fermentation cultures treated with tween 40 during the logarithmic phase exhibited a significant improvement in l-ornithine production, which is consistent with previous work which reported that addition of tween 40 promoted the production of l-glutamate [28, 29] and l-arginine [30] in *C. glutamicum*. However, addition of tween 40 in the incipient fermentation period (as early as eight hours after the start of fermentation) resulted in a remarkable decline of the l-ornithine production titer that was likely due to the growth inhibition of tween 40, which was also reported in other *C. glutamicum* strains [31]. Our observations suggest that the addition of tween 40 exerts a positive effect on l-ornithine accumulation, which provides an effective approach to reducing the production cost of l-ornithine. To further investigate the physical mechanism triggered in *C. glutamicum* Orn8 by adding the appropriate amount of tween 40, a total quantitative proteomics assay was performed and 180 proteins were discovered that exceeded a 1.2-fold expression change. Bioinformatics analysis of these proteins suggested that the overexpression of the thiamine synthesis pathway, attenuation of fatty acid synthesis pathway, and reduction of the biotin uptake system were putatively correlated with the improvement of the l-ornithine production titer induced by tween 40 addition. Thiamine is a component of the pyruvate dehydrogenase complex and converts pyruvate to acetyl-CoA, which is widely used for the production of various products including l-valine, 2-ketosiovalerate, pyruvate, succinate, and isobutanol [32]. Previous studies have identified Acetyl-CoA, an important cofactor, as a rate-limiting step for l-ornithine production by the deletion of the acetate biosynthesis pathway and by enhancing the glycolytic pathway [17]. Thus, it is generally accepted that the addition of tween 40 stimulates the supply of intracellular acetyl-CoA by upregulating the thiamine synthesis pathway, thereby increasing the production of l-ornithine. In addition, the reduced expression of acetyl-CoA carboxylase, a biotin dependent enzyme encoded by *dtsR1* and *dtsR2*, indicated that the biosynthesis of fatty acids was inhibited by tween 40 addition, which presumably leads to cell wall becomes more permeable by reducing the mycolic acid layer and provide abundant acetyl-CoA for the production of l-ornithine. It has been established that attenuating the expression of *dtsR1* was processed by tween 40 addition, which was consistent with previous work performed in *C. crenatum* which discovered that the addition of tween 40 inhibited the expression of *dtsR1* and stimulated l-arginine overproduction [24]. Furthermore, biotin is a cofactor of acetyl-CoA carboxylase involve in the biosynthesis of fatty acids. The excessive supply of biotin frequently leads to the production of a large amount of fatty acids which improved the thickness of the cell wall and hampered the secretion of glutamate [33, 34]. As discussed in previous studies, it was claimed that the addition of tween 40 or penicillin was able to change the rigid cell wall created by excess biotin via downregulation of the expression of *dtsR1*, attenuating α-oxoglutarate dehydrogenase activity, and inhibiting the biosynthesis of peptidoglycan which showed beneficial influences on the promotion of l-glutamate accumulation [35]. In addition to the reduced expression of *dtsR1*, this study also found that the biotin uptake system composed of the *bioYMN* operon was downregulated by tween 40 addition, which indicated that l-glutamate production that was induced by tween 40 under conditions with excessive biotin was probably due to the attenuation of the biotin uptake system.

To detect the presence of other unknown proteins associated with increased ornithine production, the effect of overexpressed proteins, which were upregulated by more than two-fold by tween 40 addition, on l-ornithine production was also examined in strain *C. glutamicum* Orn8. Overexpression of a putative ABC transporter, encoded by *CgS9114_09558,* which shows 3.3-fold upregulated expression, exerts a positive effect on l-ornithine accumulation, illustrating that this membrane is able to directly or indirectly contribute to the biosynthesis of l-ornithine. This observation reveals a novel transporter involved in l-ornithine production. However, Overexpression of the other genes, more than two-fold upregulated by tween 40 addition, was toxic to the cell growth, which is consist with the significant reduction of OD_600_ and l-ornithine production titer induced by addition of tween 40 prior to 6 h (Fig. 1). Those results further confirmed our previous work indicated that addition of tween 40 caused toxic effect on the cell growth of strain *C. crenatum* [24]. Notably, modulation of those genes with the highest expression change folds by tween 40 addition suggested that deleting *CgS9114_13845,* which encodes the phage peptidoglycan binding endopeptidase, *CgS9114_02593* that encodes a lipocalin, and *CgS9114_02058,* which encodes an l, d-transpeptidase associated with the biosynthesis of cell wall, could promote the biosynthesis of l-ornithine. It can be speculated that modulating the expression of genes involved in cell wall formation enhanced the production titer of l-ornithine, which is consistent with a previous report that the inhabitation of transpeptidase caused by the addition of penicillin resulted in the improvement of cell permeability and l-glutamate overproduction [27].

## Conclusion

The addition of fermentation triggers has been extensively studied for its ability to promote the production of various valuable compounds. This study, for the first time, examined the effects of tween 40 addition on l-ornithine production and evaluated the fundamental mechanisms responsible for l-ornithine overproduction in *C. glutamicum*. Improvement of the thiamine biosynthesis pathway and attenuation of the biotin uptake system discovered using a total proteomics analysis were putatively associated with the increased production of l-ornithine. Interestingly, overexpression of *CgS9114_09558* and deletion of *CgS9114_13845*, *CgS9114_02593*, and *CgS9114_02058*, proteins with higher expression multiples derived from the total proteomics analysis, further improved l-ornithine production in *C. glutamicum* Orn8. This study provides new support for the hypothesis that tween 40 addition can be used for the inducement of amino acid production. *C. glutamicum* is an excellent model organism for producing l-glutamate and l-glutamate-derived products including l-ornithine, -citrulline, and l-arginine. We believe that the metabolic engineering strategies reported in this work can be applied to constructing strains that will efficiently generate these products.

## Methods

### Strains and plasmids

*Corynebacterium glutamicum* Orn8, a l-ornithine producing strain constructed in our previous study [18, 36], derived from *C. glutamicum* S9114 was applied for strain development. Engineered strains and the plasmids used and developed in this study are listed in Table 3. For gene cloning and manipulation, *E. coli* DH5α was employed as the host. Common mediums such as LB, LBG, LBHIS and et al. were used to genetic engineering of *C. glutamicum*.

**Table 3:** Strains and plasmids used in this study

Strain/plasmid	Characteristic	Source
Strain		
*E. coli* DH5ɑ	Clone host strain	Transgen
Orn8	*C. glutamicum* S9114 with *deletion of argF, ncgl1221, argR, putP, attenuation of odhA,* and *overexpression of lysE*	Lab stock
Orn17	Orn8 carrying expression vector pEC-XK99E	This study
Orn18	Orn8 carrying expression vector pEC-*CgS09558*	This study
Orn19	Orn8 carrying expression vector pEC-*CgS14252*	This study
Orn20	Orn8 with P_*tac*_ promoter inserted in front of *CgS9114_09558*	This study
Orn21	Orn8 with P_*tac*_ promoter inserted in front of *CgS9114_14252*	This study
Orn22	Orn8 with deletion of *CgS9114_13845*	This study
Orn23	Orn8 with deletion of *CgS9114_02593*	This study
Orn24	Orn8 with deletion of *CgS9114_02058*	This study
Orn25	Orn8 with deletion of *CgS9114_14352*	This study
Orn26	Orn8 with deletion of *CgS9114_04952*	This study
Orn27	Orn8 with deletion of *CgS9114_14497*	This study
Orn28	Orn8 with deletion of *CgS9114_13845* and *CgS9114_02593*	This study
Orn29	Orn8 with deletion of *CgS9114_13845*, *CgS9114_02593* and *CgS9114_02058*	This study
Orn30	Orn8 with deletion of *CgS9114_13845*, *CgS9114_02593*, and *CgS9114_02058*; overexpression of *CgS9114_09558*	This study
Plasmid		
pK18*mobsacB*	Mobilizable vector, allows for selection of double crossover in *C. glutamicum*, Km^R^, *sacB*	[16]
pEC-XK99E	A shuttle expression vector, Km^R^	Lab stock
pEC-*CgS14252*	A derivative of pEC-XK99E, harboring *CgS9114_14252* gene from *C. glutamicum* S9114 under P_*eftu*_ promoter	This study
pEC-*CgS09558*	A derivative of pEC-XK99E, harboring *CgS9114_09558* gene from *C. glutamicum* S9114 under P_*eftu*_ promoter	This study
pK18-P_*tac*_-*CgS09558*	A derivative of pK18*mobsacB*, harboring P_*tac*_-*CgS09558* fragment	This study
pK18-P_*tac*_-*CgS14252*	A derivative of pK18*mobsacB*, harboring P_*tac*_-*CgS14252* fragment	This study
pK18-Δ*CgS13845*	A derivative of pK18*mobsacB*, harboring Δ*CgS9114_13845* fragment	This study
pK18-Δ*CgS02593*	A derivative of pK18*mobsacB*, harboring Δ*CgS9114_02593* fragment	This study
pK18-Δ*CgS02058*	A derivative of pK18*mobsacB*, harboring Δ*CgS9114_02058* fragment	This study
pK18-Δ*CgS14352*	A derivative of pK18*mobsacB*, harboring Δ*CgS9114_14352* fragment	This study
pK18-Δ*CgS04952*	A derivative of pK18*mobsacB*, harboring Δ*CgS9114_04952* fragment	This study
pK18-Δ*CgS14497*	A derivative of pK18*mobsacB*, harboring Δ*CgS9114_14497* fragment	This study

Superscript ‘‘R’’ indicates resistance to the following antibiotics: Km kanamycin

### Strain cultivation and shake flask fermentation

For the cultivation of engineered strains and shake flask fermentations, a bath culture method was performed as described previously [15, 16]. Polyoxyethylene sorbitan monopalmitate (Tween 40) was added to the shake flask in the fermentation prophase.

### Protein extraction and trypsin digestion

After being treated with tween 40 for 4 h, cells were collected at 12 h by centrifugation (5000×*g* at 4 °C for 10 min). Cells without tween 40 treatment were used as a control. Cell pellets were washed twice using cold de-ionized water. Subsequently, samples were resuspended in lysis buffer, containing 8 M urea and 1% protease inhibitor cocktail, and then the turbid liquid was sonicated three times using a high intensity ultrasonic processor (Scientz). The residual debris was removed by centrifugation (12,000×*g* at 4 °C for 10 min). After centrifugation, the lysate supernatant was identified as the crude protein sample and the protein concentration was determined using a BCA kit according to the manufacturer’s instructions.

An appropriate amount of protein solution was used for trypsin digestion. At first, the protein solution was diluted to 5 mM by using dithiothreitol and incubated at 56 °C for 30 min. Then, the solution was alkylated with 11 mM iodoacetamide for 15 min and protected from light and held at room temperature. Furthermore, the protein solution was diluted to a urea concentration of less than 2 M by adding the appropriate amount of triethylamine borane. Finally, trypsin was added at a trypsin-to-protein mass ratio of 1:50 for the first overnight-digestion and with a trypsin-to-protein mass ratio of 1:100 for a second 4 h-digestion.

### Peptide labeling

After trypsin digestion, the randomly interrupted peptide fragments were desalted using a Strata X C18 SPE column (Phenomenex) and vacuum dried. Peptide fragments were resuspended in a 0.5 M triethylamine borane solution and labeled using a TMT kit according to the manufacturer’s protocol. Next, the thawed TMT reagent was briefly reconstituted in acetonitrile solution. Then, the TMT solution and peptide fragment mixtures were incubated for 2 h at room temperature, desalted, and dried by vacuum freezing.

### HPLC fractionation and LC–MS/MS analysis

High pH reverse-phase HPLC using an Agilent 300Extend C18 column (5 μm particles, 4.6 mm ID, 250 mm length) was employed for the fractionation of tryptic peptides fractions. Peptides were first separated into 60 fractions using acetonitrile (pH 9.0) with a gradient of 8% to 32% over 60 min. Subsequently, the peptides were mixed into 18 fractions and dried by vacuum freezing.

After dissolving them in 0.1% formic acid (solvent A), the peptide fractions were separated using an EASY-nLC 1000 UPLC system. Then, the peptides were injected into NSI ion source for ionization, followed by tandem mass spectrometry (MS/MS) analysis in Orbitrap FusionTM TribridTM (Thermo).

### Database search and bioinformatics analysis

The resulting MS/MS data were processed using the Maxquant search engine (v.1.5.2.8). Tandem mass spectra were searched against the UniProt *C. glutamicum* database. A reverse decoy database search strategy was employed to evaluate the fault rate for peptide identification. The specific parameters were listed as follows: (1) enzyme, trypsin allowing up to 2 missing cleavages; (2) protein level FDR ≤ 1%; (3) peptide length ≥ 7 aa; (4) unique peptides ≥ 2; (5) contaminants, trypsin; (6) protein modification. Carbamidomethyl on Cys was specified as a fixed modification and oxidation on Met was specified as a variable modification. Subsequently, proteins with an average ratio > 1.2-fold increase or decrease were confidently considered as the differentially expressed proteins. Gene Ontology (GO) annotation of the total proteins was derived from the UniProt-GOA database (www. http://www.ebi.ac.uk/GOA/). If some identified proteins were not annotated by the UniProt-GOA database, the InterProScan software was employed for GO annotation of those proteins. The Kyoto Encyclopedia of Genes and Genomes (KEGG) database was used to annotate and identify enriched pathways. These pathways were classified into hierarchical categories according to the KEGG website. We first collated all the categories obtained after enrichment along with their P values, and then filtered for those categories which were at least enriched in one of the clusters with a P value < 0.05. This filtered P value matrix was transformed by the function x = − log10 (P value).

### DNA manipulation and strain construction

For the genetic engineering of genes in *C. glutamicum*, a suicide vector pK18*mobsacB* and expression plasmid pEC-XK99E based standard method was performed as described previously [37–39]. The primers used in this work are listed in Additional file 3: Table S3. 

### Measurement of cell growth and metabolites concentration

The biomass was monitored by measuring the OD_600_ using a microplate reader (BioTek Instruments, Winooski, VT, USA) after dissolving CaCO_3_ in 0.125 mol/L HCl. The l-ornithine titer was measured by colorimetry using ninhydrin as described previously [40, 41]. An SBA-40C biosensor (developed by Biology Institute of Shandong Academy of Sciences) was employed for glucose analysis in fermentation liquid [42]. To increase the reliability of measurements, samples were collected from three parallel experiments in order to calculate average values and standard deviations.

## Supplementary information

**Additional file 1: Table S1.** The profiles of whole protein information identified by MS.

**Additional file 2: Table S2.** Differentially expressed protein statistics induced by tween 40 addition.

**Additional file 3: Table S3.** The profiles of all primers used in this study.

## Data Availability

Gene sequences used in this project are from Genbank (http://www.ncbi.nlm.nih.gov/) and the material and data supporting their findings can be found in the main paper and the additional file.
