# Supplementary material for: Proteome analysis guided genetic engineering of Corynebacterium glutamicum S9114 for tween 40-triggered improvement in l-ornithine production
Source: Microb Cell Fact. 2020 Jan 6;19:2. doi: 10.1186/s12934-019-1272-0 (PMC6943917; doi:10.1186/s12934-019-1272-0)
Supplement: Supplementary file 3 — Additional file 3: Table S3. The profiles of all primers used in this study. [file 12934_2019_1272_MOESM3_ESM.docx]

TABLE S3 Primers and their sequences in this study

| Primers | Sequence (5’-3’) |
| --- | --- |
| *P_eftu_*-F: | cacaggaaacagaccatggaattcCACAGGGTAGCTGGTAGTTTGAA |
| *P_eftu_*-R: | TGTATGTCCTCCTGGACTTCGTG |
| *CgS9114_09558*-F(pEC): | CACGAAGTCCAGGAGGACATACAATGACAACGACACCAGCAATCGAC |
| *CgS9114_09558*-R(pEC): | gactctagaggatccccgggtaccCTAGGCGGTTTGCAGCGAGGTGTT |
| *CgS9114_14252*-F(pEC): | CACGAAGTCCAGGAGGACATACAATGGCTAGTCCTGCCATGCAA |
| *CgS9114_14252*-R(pEC): | gactctagaggatccccgggtaccTTATACCTCGCGGCCAGC |
| *CgS9114_09558*-up-F: | aacgacggccagtgccaagctGCGGTTCTATAACTTGGCGTTT |
| *CgS9114_09558*-up-R(tac): | TAACCATTATAACACAGATTCAAATTAATGTCAACCCGGCATAATTCTGCCCATTCCG |
| *CgS9114_09558*-down-F(tac): | TTTGAATCTGTGTTATAATGGTTAAGGAGATATACATGACAACGACACCAGCAATCGAC |
| *CgS9114_09558*-down-R: | cggtacccggggatcctctagCTAGGCGGTTTGCAGCGAGGTGTT |
| *CgS9114_14252*-up-F: | aacgacggccagtgccaagctGCGCTCATTGTTGATGGAGAT |
| *CgS9114_14252*-up-R(tac): | TAACCATTATAACACAGATTCAAATTAATGTCAACCCGCATTATCCGGATCAGGTTCG |
| *CgS9114_14252*-down-F(tac): | TTTGAATCTGTGTTATAATGGTTAAGGAGATATACATGGCTAGTCCTGCCATGCAA |
| *CgS9114_14252*-down-R: | cggtacccggggatcctctagTTATACCTCGCGGCCAGC |
| *CgS9114_13845*-up-F： | aacgacggccagtgccaagcttCGATAAGCCATTCTGGAGCAC |
| *CgS9114_13845*-up-R： | AAAAGGGCGATCATCTGACCGCCCTTGCCACAAAACTTTTCGAAG |
| *CgS9114_13845*-down-F： | GGGCGGTCAGATGATCGCCCTTTTTTTTTAAGCAGGCGAAATTGCAGAAG |
| *CgS9114_13845*-down-R： | cggtacccggggatcctctagaGAGCATGAGCACCAAGAGTGC |
| *CgS9114_02593*-up-F： | aacgacggccagtgccaagcttCGCAAATTCATACGACTTTCC |
| *CgS9114_02593*-up-R： | AAAAGGGCGATCATCTGACCGCCCTAGTGCTGTGGTGACAAGTTTGC |
| *CgS9114_02593*-down-F： | GGGCGGTCAGATGATCGCCCTTTTTTTTTTCGGGATATCCACGGCACAA |
| *CgS9114_02593*-down-R： | cggtacccggggatcctctagaCTTTCGCCCTCATGGTCACTG |
| *CgS9114_**02058*-up-F： | aacgacggccagtgccaagcttAGACGTGGAAGCATGGGTTTC |
| *CgS9114_02058*-up-R： | AAAAGGGCGATCATCTGACCGCCCCGCTGTATCGGATTCGAGAA |
| *CgS9114_02058*-down-F： | GGGCGGTCAGATGATCGCCCTTTTTTTTTTTGATCGCTGTGCCAGCAACT |
| *CgS9114_02058*-down-R： | cggtacccggggatcctctagaTCCTCCGCTTTTCCAGAACAC |
| *CgS9114_**14352*-up-F： | aacgacggccagtgccaagcttGTGCAGATGCGAGCTCAAGAA |
| *CgS9114_14352*-up-R： | AAAAGGGCGATCATCTGACCGCCCGGGGAGTGTCTTTGTCACTTG |
| *CgS9114_14352*-down-F： | GGGCGGTCAGATGATCGCCCTTTTTTTTTCGGCTGTCACACCAGTTGAAC |
| *CgS9114_14352*-down-R： | cggtacccggggatcctctagaACGCAATCCAAGTTCTGGGAG |
| *CgS9114_04952*-up-F： | aacgacggccagtgccaagcttTGCTGACATCGCTGTGGAGGT |
| *CgS9114_04952*-up-R： | AAAAGGGCGATCATCTGACCGCCCTTCATACGTCTTTCCCTCGTG |
| *CgS9114_04952*-down-F： | GGGCGGTCAGATGATCGCCCTTTTTTTTTGGAATTGCGATGTCCACCATT |
| *CgS9114_04952*-down-R： | cggtacccggggatcctctagaTGGTTGAGTCCTTCGAAGCGT |
| *CgS9114_14497*-up-F： | aacgacggccagtgccaagcttCACTGCCACTGGTAGAAAACC |
| *CgS9114_14497*-up-R： | AAAAGGGCGATCATCTGACCGCCCCTCGCACCTTTTGGGGAACTG |
| *CgS9114_14497*-down-F： | GGGCGGTCAGATGATCGCCCTTTTTTTTTAACATGGCTCAGCGAAAACTG |
| *CgS9114_14497*-down-R： | cggtacccggggatcctctagaTTGACCCAAGGATCATTGGAG |

Restriction enzyme sites were represented as bold characters. The overlapping region with PCR fragment were marked by underline. The overlapping region with vector was represented as lowercase.
